# Unveiling the ferrielectric nature of PbZrO_3_-based antiferroelectric materials

**DOI:** 10.1038/s41467-020-17664-w

**Published:** 2020-07-30

**Authors:** Zhengqian Fu, Xuefeng Chen, Zhenqin Li, Tengfei Hu, Linlin Zhang, Ping Lu, Shujun Zhang, Genshui Wang, Xianlin Dong, Fangfang Xu

**Affiliations:** 1 State Key Laboratory of High Performance Ceramics and Superfine Microstructures, Shanghai Institute of Ceramics, Chinese Academy of Sciences, 200050 Shanghai, China; 2 The Key Lab of Inorganic Functional Materials and Devices, Shanghai Institute of Ceramics, Chinese Academy of Sciences, 200050 Shanghai, China; 3 University of Chinese Academy of Sciences, 100049 Beijing, China; 4 School of Physical Science and Technology, ShanghaiTech University, 201210 Shanghai, China; 5 Institute for Superconducting and Electronic Materials, Australian Institute of Innovative Materials, University of Wollongong, Wollongong, NSW 2500 Australia

**Keywords:** Ferroelectrics and multiferroics, Phase transitions and critical phenomena

## Abstract

Benefitting from the reversible phase transition between antiferroelectric and ferroelectric states, antiferroelectric materials have recently received widespread attentions for energy storage applications. Antiferroelectric configuration with specific antiparallel dipoles has been used to establish antiferroelectric theories and understand its characteristic behaviors. Here, we report that the so-called antiferroelectric (Pb,La)(Zr,Sn,Ti)O_3_ system is actually ferrielectric in nature. We demonstrate different ferrielectric configurations, which consists of ferroelectric ordering segments with either magnitude or angle modulation of dipoles. The ferrielectric configurations are mainly contributed from the coupling between A-cations and O-anions, and their displacement behavior is dependent largely on the chemical doping. Of particular significance is that the width and net polarization of ferroelectric ordering segments can be tailored by composition, which is linearly related to the key electrical characteristics, including switching field, remanent polarization and dielectric constant. These findings provide opportunities for comprehending structure-property correlation, developing antiferroelectric/ferrielectric theories and designing novel ferroic materials.

## Abstract (web summary)

The large family PbZrO_3_-based solid solutions are usually considered as antiferroelectric materials with specific antiparallel polarization configuration. Here, the authors demonstrate the PbZrO_3_-based material has ferrielectric dipoles ordering and configure a clear structure-property relationship.

## Introduction

Dielectric capacitors have been commercialized for a wide range of applications including pulsed power systems, electrical vehicles, and medical devices^1–4^. Benefitting from the electrical field induced reversible phase transition between antiferroelectric (AFE) and ferroelectric (FE) states, AFE perovskite oxides (ABO_3_) become promising candidate for dielectric energy storage capacitors due to their high power density, good energy storage density, and long lifetime^5–8^.

Early in 1951, Kittel theoretically defined AFE configuration in analogy with antiferromagnetism as neighboring lines of dipoles pointing in antiparallel directions, while Sawaguchi et al. experimentally observed AFE behavior in PbZrO_3_ ceramic according to its characteristic dielectric response and hysteresis loop^9,10^. Later on, the X-ray diffraction, neutron diffraction, convergent-beam electron diffraction, and high-resolution transmission electron microscopy have been applied to substantiate the AFE configuration in PbZrO_3_^11–15^. Based on these comprehensive structural investigations, the basic dipoles ordering configuration of PbZrO_3_ was established in which the two left-oriented dipole lines and two right-oriented dipole lines arrange alternately. Therefore, the PbZrO_3_ has become the prototypical AFE material for understanding the physics of AFE and exploring new AFE materials. For AFE physics, theoretical models always involve ferrielectric (FiE) phase when AFE order parameter is coupled with field-induced polarization^16,17^, while the FiE configuration has never been experimentally established. For AFE materials, numerous chemical elements (A-sites: La^3+^, Ba^2+^, Ca^2+^, Sr^2+^, etc.; B-sites: Sn^4+^, Ti^4+^, Nb^5+^, etc.) have been attempted in PbZrO_3_ for decreasing its extremely high switching field and optimizing energy storage characteristics^18–21^. As a consequence, the large family PbZrO_3_-based solid solutions were considered as AFE materials with specific antiparallel polarization configuration, which has been rarely challenged.

In PbZrO_3_-based ceramics, the chemical doping generally drives the AFE phase from commensurate structure to incommensurately modulated structure (IMS). The complex IMS was firstly investigated by MacLaren et al. in (Pb_0.96_La_0.04_)(Zr_0.9_Ti_0.1_)_0.99_O_3_ ceramics on atomic-scale, where the modulation of A-site cations was found to exist in a sinusoidal fashion^22^. Ma et al. further pointed out that the A-site cations were either antiparallel but imbalanced or in a nearly orthogonal arrangement in (Pb_0.99_Nb_0.02_[(Zr_0.57_Sn_0.43_)_1−y_Ti_y_]_0.98_O_3_ ceramics^23^. These works have been regarded as an extraordinary step forward to the understanding of cation displacement in IMS. Nonetheless, early work has demonstrated that the prototypical PbZrO_3_ showed multiple soft-mode vibrations, which mainly involved trilinear coupling between vibrations of Pb cations and librations of oxygen octahedra^24^. Moreover, the very recent work on the electron-beam illumination induced phase transition in PbZrO_3_ single crystals clearly showed the hierarchical evolution of oxygen octahedra^25^. Thus, as an important component in perovskite PbZrO_3_-based materials, the information of oxygen octahedra should be essentially taken into account for comprehending structure-property correlation and developing AFE/FiE theories.

Here, we use atomic-resolved scanning transmission electron microscopy to explore the electric dipoles ordering configurations in (Pb,La)(Zr,Sn,Ti)O_3_ (PLZST) system, which is one of the most promising PbZrO_3_-based AFE materials. We demonstrate the so-called AFE PLZST system has actual arrangement of dipoles in FiE configuration. The coupling between A/B-cations and O-anions is explicitly elucidated. Based on the FiE configurations, the key electrical properties can be clearly understood upon varying the composition.

## Results

### Characterization of average structure

**Fig. 1:**
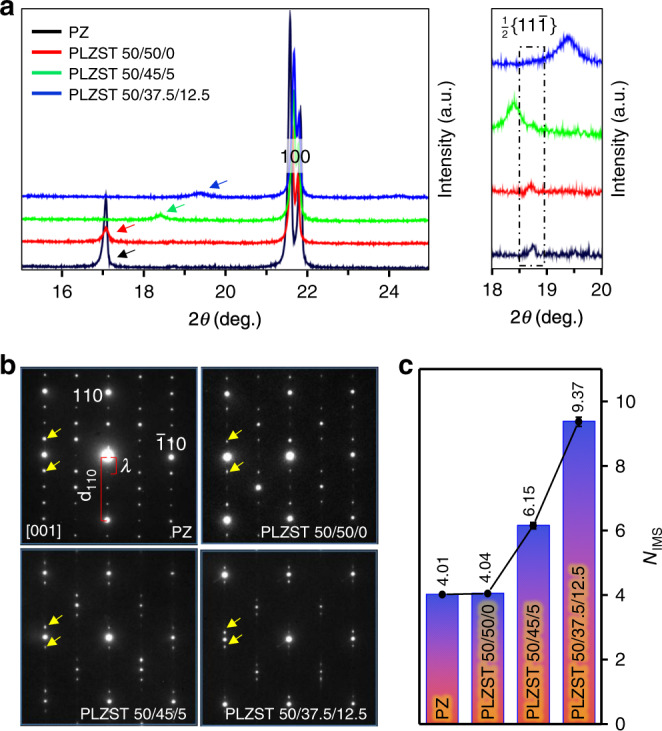
Average structural characteristics of PbZrO_3_ and PLZST system. **a** The XRD patterns showing two types of superlattice reflections originated from the cations modulation marked by arrows and octahedra tilts marked by dash rectangle, respectively. The 2θ range of 18°–20° are enlarged on the right to clearly show 1/2{11$\bar 1$} peaks associated with antiphase tilt of oxygen octahedra. The main {100} peaks of PbZrO_3_ is approximately aligned with PLZST 50/50/0 for clearly comparing modulation period. **b** The [001] SAED patterns showing the modulation wave along [110] direction. The satellite reflections associated with modulation mode are marked by yellow arrows. **c** The modulation period (*N*_IMS_) = *λ*/d_110_ (*λ* refers to modulation wavelength in real-space) calculated from SAED patterns. The average data of modulation period for each sample is obtained from more than five different areas and error bars represent the standard deviation.

Pb_0.97_La_0.02_(Zr_0.50_Sn_x_Ti_0.50-x_)O_3_ ceramics with *x* = 0.50, 0.45, 0.375 (hereafter denoted as PLZST 50/50/0, PLZST 50/45/5, and PLZST 50/37.5/12.5, respectively) were prepared by high temperature solid-state sintering method. X-ray diffraction studies reveal their crystal structures are periodically modulated because the characteristic superlattice reflections (marked by arrows) appear ahead of the {100} basic reflection (Fig. 1a, all crystallographic indices refer to the simple pseudocubic unit cell in this work). According to the crystal structure of PbZrO_3_, these peaks are mainly associated with the displacement of cations. They gradually move upward to the {100} peaks indicating the modulation period (*N*_IMS_) increases with decreasing Sn/Ti ratio. Combined with the selected area electron diffraction (SAED) patterns of [001], [100], and [1$\bar 1$2] zone axes (Fig. 1b and Supplementary Fig. 1), it is confirmed that the modulation wave is along [110] direction with average *N*_IMS_ of 4.04, 6.15, and 9.37 for PLZST 50/50/0, PLZST 50/45/5, and PLZST 50/37.5/12.5, respectively (Fig. 1c). The fractional (irrational) values of *N*_IMS_ indicate that all three PLZST compositions show IMS. Meanwhile, the appearance of characteristic superlattice reflections associated with oxygen octahedral tilts in XRD (Fig. 1a, marked by dash rectangle) and [1-1.2] SAED patterns (Supplementary Fig. 1a, marked by blue arrow) indicates that the octahedra of PLZST have the similar antiphase tilt with PbZrO_3_. It can be seen that the intensity of 1/2{11$\bar 1$} reflections gradually decreases with decreasing Sn/Ti ratio. This implies the strong coupling of octahedra tilts with cations displacement, which is believed to stabilize the modulated structure^24,26,27^ and will be further discussed in the following.

### Atomic-scale analysis of modulated structure

**Fig. 2:**
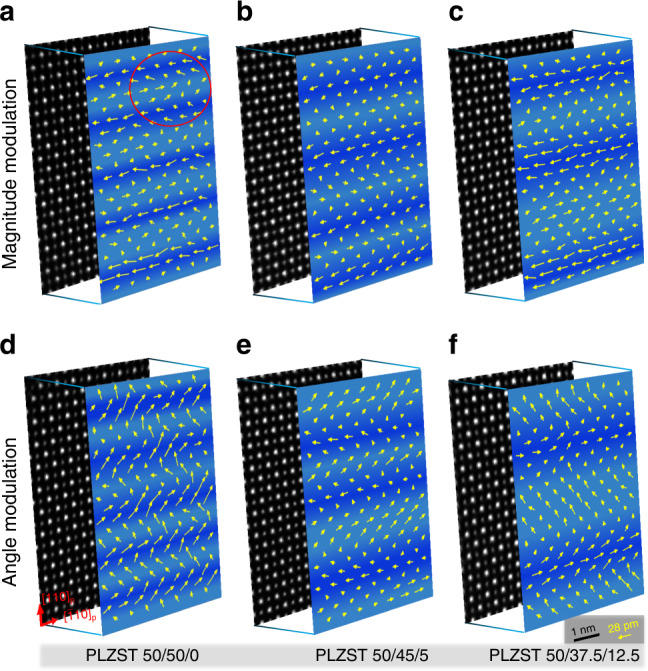
Polarization mapping based on cations displacement in PLZST system. **a–c** Magnitude modulation mode and (**d–f**) angle modulation mode of dipoles ordering configurations (front) as revealed from the atomic-scale HAADF images (back) acquired along the [001] direction. The dark/light-blue backgrounds highlight the switch of polarization configuration. The full view of the corresponding HAADF images are presented in Supplementary Fig. 2. The red circled area in (**a**) shows the dipoles ordering configuration being nearly consistent with the one in PbZrO_3_.

Because spontaneous polarization (**P**_**s**_) linearly relates with the atomic displacement, atomic-scale high-angle annular dark field (HAADF) imaging was performed to investigate the dipoles ordering configurations based on cations displacement in PLZST system. The picometer-precision fitting was applied to extract the cation column positions (see the details in Methods). Previous neutron diffraction studies on Zr-rich Pb(Zr,Ti)O_3_ confirmed that Pb cations were responsible for the spontaneous polarization because the displacement magnitude of Pb cations was about three times that of Zr/Ti^28,29^. Similarly, it can be immediately accessible by directly observing the experimental HAADF images (Supplementary Fig. 2) that the A-site cations have obviously larger displacement than B-site cations in our PLZST system. Thus, we define the atomic displacement vectors (**D**_**AB**_) as the relative offset of the A-site cations from the center of four adjacent B-site cations in the (001) plane. Displacement maps (Fig. 2) reveal that the dipoles are modulated periodically in all PLZST compositions, leading to the appearance of coherent fringes in medium-magnified TEM images (Supplementary Fig. 3). The modulation intervals are found to increase with decreasing Sn/Ti ratio, not necessarily only two dipole lines as modeled in the prototypical AFE configuration. The modulation structures are of incommensurate-type with fluctuant periods, which could be intuitively viewed from the width of dark/light-blue segments. Interestingly, we observed two different modulation modes (either magnitude-type or angle-type) for each PLZST composition. In the magnitude modulation mode (Fig. 2a–c), all dipoles align nearly horizontally where the dark-blue segments exhibiting larger **D**_**AB**_ magnitude than the light-blue segments. On the other hand, in the angle modulation mode (Fig. 2d–f), the dipoles in the dark-blue segments and the light-blue segments have approximately equal **D**_**AB**_ magnitude but their orientations change alternately. In such case, it can be predicted that the net polarization should produce in both magnitude (in horizontal direction) and angle (in vertical direction) modulation modes. Therefore, we can conclude that the so-called AFE PLZST system essentially possesses ferrielectric (FiE) nature (also see the quantitative data in Supplementary Fig. 4). Based on the analysis for several areas in each PLZST composition, we found that the magnitude modulation mode was dominant and the two modulation modes could interchange freely (Supplementary Fig. 5). Besides, the polarization tends to be zero at the transitional area between dipole lines of different orientations (see the interface between dark-blue and light-blue segments), especially in the magnitude modulation mode. It can be noted that the PLZST 50/50/0 inherits the fourfold modulation period with some local areas approximately maintaining the dipoles ordering configuration of PbZrO_3_ (e.g., the red circled area in Fig. 2a).

**Fig. 3:**
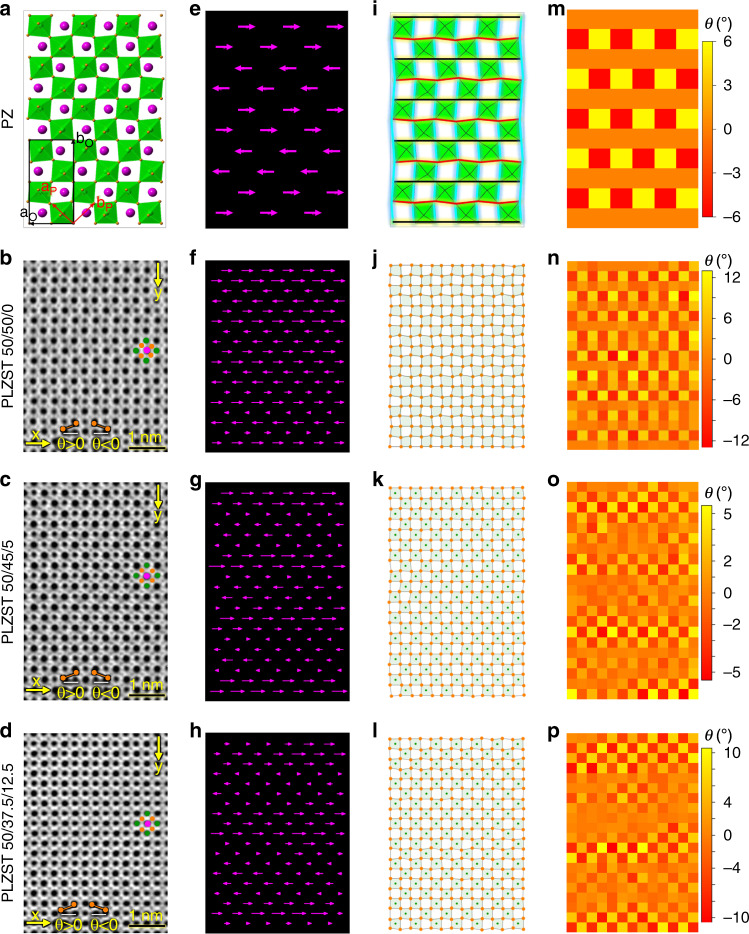
Structural mapping of the coupling between O-anions displacement and A/B-cations displacement. **a–d** The [001]_p_ projected structure of PbZrO_3_ AFE phase (ref. ^11^) and experimental ABF images of PLZST system, where purple, green, and orange balls represent A-cations, B-cations, and O-anions, respectively. The axis of “a_O_/b_O_” and “a_P_/b_P_” refer to orthorhombic and pseudocubic unit cell, respectively. The directions of “x/y” are defined as the [$\bar 1$10]_p_/[$\bar 1\bar 1$0]_p_, respectively. **e**–**h**, (**i–l**), and (**m–p**) are the corresponding mappings of relative cations displacement (**D**_**AB**_), tilts and distortion of BO_6_ octahedra, and y-rippling of O-sublattice, respectively. The black/red/cyan lines in (**i**) highlight the displacement behaviors of O-anions along x- and y-direction in PbZrO_3_. The (**e–h**) only show the horizontal component of **D**_**AB**_ for clarity. The color scales represent local amplitude of y-rippling of O-sublattice, which is defined as Ɵ, i.e., the angle between O–O bond in a row and horizontal x-direction. The mapping of x-rippling of O-sublattice is not shown because it can be accessible in the corresponding quantitative profiles presented in Fig. 4.

**Fig. 4:**
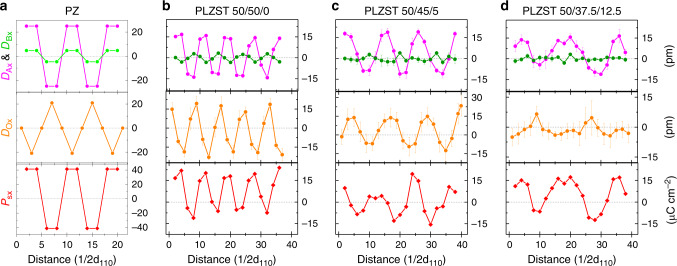
Quantitative analysis of coupling between O-anions displacement and A/B-cations displacement. **a–d** The horizontal component of atomic displacement and spontaneous polarization for each atomic row is plotted as a function of distance along the y-direction for PbZrO_3_ and PLZST system. The purple, green, orange, and red profiles represent the A-cations, B-cations, O-anions, and *P*_*s*_, respectively. The value of these profiles are calculated from the Fig. 3a–d, where the displacement are defined as negative value when it aligns to the left. The corresponding profiles for vertical component are presented in Supplementary Fig. 8. The error bars represent the standard deviation measured from the experimental image.

In order to explore the oxygen octahedral contribution to the observed FiE configuration and understand the coupling between O-anions displacement and A/B-cations displacement, atomic-scale annular bright field (ABF) imaging was applied to analyze the local structural characteristic of O-sublattice in the dominant modulation mode (Figs. 3 and 4). Similarly, the **D**_**AB**_ mappings (Fig. 3e–h) clearly show modulation of cation displacement as those observed in HAADF images (Fig. 2). It is generally accepted that the equally antiparallel **D**_**AB**_ collaborates with antiphase tilting of oxygen octahedra to stabilize the prototypical AFE structure in PbZrO_3_. Actually, the distortion of oxygen octahedra should also have an important influence on the prototypical AFE structure, which can be clearly seen from the irregular shape of oxygen octahedra in Fig. 3a. The tilt and distortion of oxygen octahedra can be characterized by measuring the rippling of O-sublattice along x- and y-direction (Fig. 3i). Based on the experimental ABF images (Fig. 3b–d), we map the corner-shared BO_6_ octahedra of PLZST system (Fig. 3j–l). The as-expected tilt and distortion of oxygen octahedra (see the irregular shape of oxygen octahedra and the rippling of O-row/column) was then found to occur simultaneously contributing to the observed FiE configuration in PLZST system. Interestingly, the coupling along x-direction is manifested by the modulation of y-rippling for O-sublattice, i.e., the large/small magnitude of **D**_**AB**_ usually corresponds to the high/low degree of y-rippling of O-rows (Fig. 3f–h, n–p), which can be understood by the Coulomb interaction (Supplementary Fig. 6). Thus, it can be understood that the decrease of intensity of 1/2{11$\bar 1$} reflections in XRD (Fig. 1a) and SAED (Supplementary Fig. 1a) is caused by the increase of volume fraction of the areas with small y-rippling amplitude. Compared with the one in PbZrO_3_ (Fig. 3m), it can be clearly seen that the y-rippling of O-rows in PLZST system breaks the long-rang antiphase octahedral tilt of a^−^a^−^c^0^ system and is periodically modulated with local disorder.

Similarly, the coupling along y-direction can be manifested by the modulation of horizontal component of atomic displacement (Fig. 4). In PbZrO_3_, the coupling features the characteristic that Zr-cations move in the same direction as Pb-cations while O-anions move in the opposite direction (Fig. 4a). In contrast, the coupling behavior in PLZST is strongly dependent on composition although displacement of both A/B-cation and O-anion are modulated in corresponding period. Specifically, firstly, the PLZST 50/50/0 has a similar way to PbZrO_3_, but there is a phase difference about one d_110_ between A-cations and B-cations/O-anions (Fig. 4b). The horizontal displacement of O-anions in Fig. 4b refers to the data of odd column while its comparison to the displacement of even column and the sum of both columns are presented in Supplementary Fig. 7. Secondly, the PLZST 50/45/5 shows inverse behavior to PbZrO_3_, i.e., B-cations move in the opposite direction to Pb-cations while O-anions move in accordance with A-site cations, synchronous alternation of large positive value and small negative value (Fig. 4c). Thirdly, the PLZST 50/37.5/12.5 presents nearly the same fashion as PbZrO_3_, in which the displacement of O-anions has approximately equal positive and negative value (Fig. 4d). Apart from the difference in coupling behavior, the displacement amplitude of both A/B-cation and O-anion gradually decrease with decreasing of Sn/Ti ratio, consequently led to the decrease of intensity of 1/2{11$\bar 1$} reflections in XRD (Fig. 1a) and SAED (Supplementary Fig. 1) as well. Of particular interest is that the displacement of O-anion gradually changes its position with the reference of zero-line while the A-cations maintain the unbalanced displacement in all three compositions. Thus, the displacement of O-anion is also strongly affected by the doping effect of B-site elements. For instance, the stronger hybridization between B-cation and O-anion occurs with higher Ti content in B-site (Zr/Sn/Ti) composition by taking account of difference in ionic radius and electronegativity.

Accompanied with the quantitative analysis of atomic displacement, the local polarization (Fig. 4 and Supplementary Fig. 8) is calculated in terms of standard definition $P_{\it{s}} \,=\, \frac{1}{V}\mathop {\sum}\nolimits_i^{} {Z_i^ \ast }$, where *V*, *δ*_*i*_ and $Z_i^ \ast$ are the volume of unit cell under consideration, atomic displacement with respect to the ideal position, and the Born effective charge, respectively. The $Z_i^ \ast$ values of cubic PbZrO_3_ in ref. ^30^ are used for the PLZST system. Clearly, the horizontal **P**_**s**_ is also periodically modulated because of the main contribution from the displacement of A-cations and O-anions. The **P**_**s**_ of PLZST system have been substantially suppressed compared with PbZrO_3_, especially in some local areas where structural disorder decreases the amplitude of the oxygen modulation. Thus, the O-anions should be taken into consideration for quantitative characterization of the polarization modulation. Nevertheless, the **P**_**s**_ profiles also presents the FiE configuration in all three PLZST compositions, being consistent with the quantitative **D**_**AB**_ mapping.

### Structure–property correlation

**Fig. 5:**
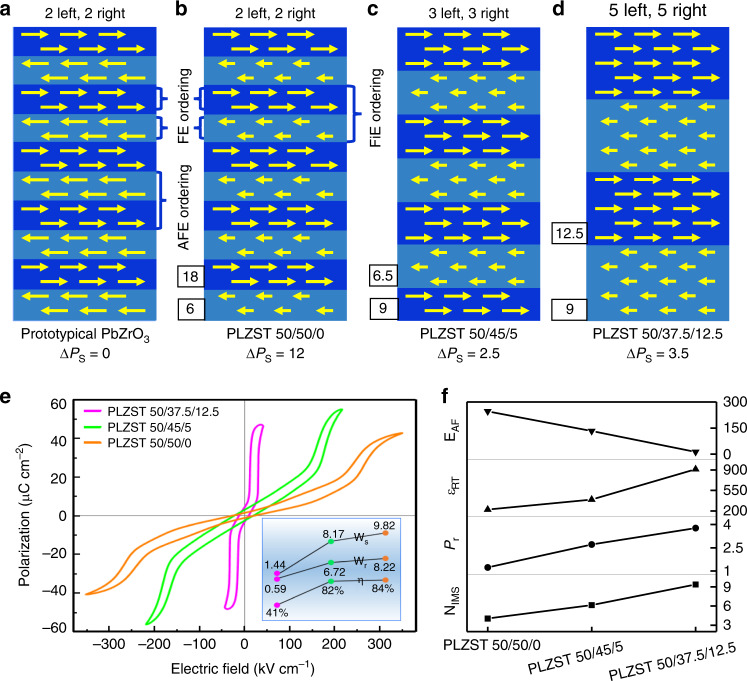
Interplay of AFE, FiE, and FE ordering and structure–property correlation. **a–d** Ideal models of dipoles ordering configurations in PbZrO_3_ and PLZST system. Arrows with left and right orientations represent the dipoles. The polarization value of FE segments in PLZST system is based on the quantitative data in Fig. 4. The ΔP_s_ refers to the net polarization of two adjacent FE segments (in unit of μC cm^−2^). **e, f** Hysteresis loops, modulation period (*N*_IMS_), remanent polarization (P_r_, μC cm^−2^), dielectric constant (ε_RT_) and forward switching field (E_AF_, kV cm^−1^) of PLZST system. The inset in (**e**) shows the energy storage density (W_s_, J cm^−3^), recoverable energy density (W_r_, J cm^−3^) and energy efficiency (η) values.

According to the above structural characterizations, the interplay of FE, FiE, and AFE orderings and the structure-property relationship can be understood comprehensively. Figure 5 models the ideal FiE ordering configurations of PLZST system compared with the AFE ordering configuration of PbZrO_3_. Here, only the dominant magnitude modulation mode is given for PLZST system. It should be noted that the dipoles ordering configurations in PLZST system actually have fluctuant periods and cyclic variation of magnitude, and in particular, the dipoles tend to be zero at the transitional area between dipole lines of different orientations (Figs. 2 and 4). Thus, the schematic illustrations with fixed modulated period and magnitude of dipoles in Fig. 5 are just the simplification of the experimental observations. Clearly, the AFE ordering originates from a couple of two-layer equivalent FE ordering segments with opposite directions (Fig. 5a) while the FiE ordering stems from two inversely polarized but nonequivalent FE ordering segments whose width is composition-dependent (Fig. 5b–d). The double/multiple hysteresis loops are so inveterate for PbZrO_3_-based materials that their AFE nature has been rarely challenged due to the similar polarization loops as observed in Fig. 5e. Nevertheless, some electric properties can hardly be understood based on prototypical AFE model. The FiE nature of PLZST system results in detectable remanent polarization (P_r_), which increases linearly with the net polarization of FE segments (ΔP_s_). It should be noted that although the measured ΔP_s_ value in the FiE region of PLZST 50/50/0 is obviously larger than the one in PLZST 50/45/5, the existence of large areas with AFE ordering (see Supplementary Fig. 9 and circled region in Fig. 2a) eventually results in an overall smaller P_r_ for PLZST 50/50/0. The modulation period (*N*_IMS_) will increase by enlarging the FE ordering segments with decreasing the Sn/Ti ratio, i.e., 2 rows, 3 rows, and 5 rows for PLZST 50/50/0, PLZST 50/45/5, and PLZST 50/37.5/12.5, respectively, due to the fact that Ti favors the ferroelectricity while Sn favors the antiferroelectricity^19^. In this case, the interactions between the nearest neighboring dipoles with opposite direction will decrease, which will lead to the increased dielectric constant (*ɛ*_RT_) and decreased forward switching field (*E*_AF_), as shown in Fig. 5f and Supplementary Table 1. We also correlated *N*_IMS_ with P_r_, ε_RT_ and E_AF_ in the Sr-doped PLZST system, where the similar trend was observed (Supplementary Fig. 10 and Supplementary Table 2), supporting the proposed correlation between modulation structure and macroscopic properties. Thus, the structure with antiparallel, equal and narrow FE segments is highly desired for high energy storage application because it has low *P*_*r*_ and large forward switching field. This has been strongly evidenced in PLZST 50/50/0, which possesses the highest energy storage density of 9.82 J/cm^3^ with satisfied energy efficiency of 84% (see the inset in Fig. 5e).

In summary, the current work determines the FiE nature of PLZST system, which has long been regarded as AFE materials. The FiE ordering originated from two unequal FE ordering segments with opposite directions (magnitude modulation) or two equal FE ordering segments with different orientations (angle modulation), which can be tailored by composition tuning, i.e., the Sn/Ti ratio in the studied PLZST system. The FiE ordering is mainly attributed to the coupling between A-cations and O-anions, which is strongly dependent on composition but has the common characteristics including, (a) both A-cations and O-anions are periodically modulated, breaking the long-range order of equally antiparallel cation displacement and antiphase octahedral tilt of the a^−^a^−^c^0^ system in PbZrO_3_; (b) the FE segments with large/small A-cations displacement correspond to the O-sublattice with high/low degree of y-rippling; (c) the tilt and distortion of oxygen octahedra simultaneously occur to stabilize the IMS. The relationship of composition, structure, and property has been established, providing a solid ground for materials design for numerous high/pulse power applications. In addition, the observation of FiE configurations is expected to motivate the exploration of AFE/FiE theories in perovskite oxides. Of particular importance is that, the chemical-driven dipole modulation, especially with large modulation period (Fig. 2f and Supplementary Fig. 5b), in PbZrO_3_-based materials may open up additional opportunities for the emerging topological domains, such as flux-closure, polar vortices, and polar skyrmions^31–33^, which up to now can only be obtained by epitaxial strain, while the coupling between epitaxial strain and chemical doping on the modulated dipole ordering configurations remains an open question.

## Methods

### Materials synthesis

Pb_0.97_La_0.02_(Zr_0.50_Sn_x_Ti_0.50-x_)O_3_ ceramics with *x* = 0.50, 0.45, 0.375 (hereafter denoted as PLZST 50/50/0, PLZST 50/45/5, and PLZST 50/37.5/12.5, respectively) were prepared by high temperature solid-state sintering method. Pb_3_O_4_, ZrO_2_, TiO_2_, La_2_O_3_, and SnO_2_ powders with the purity of at least 99.0% were weighted and 0.5 wt% excess PbO were added to compensate for the volatilization of Pb during sintering. Raw materials were ball-milled for 6 h and dried at 120 ^°^C. Then they were calcined at 900 °C for 2 h. The PLZST powders were ball-milled again for 24 h, dried, mixed with 6 wt% PVA as binding agent and then pressed into pellets of 13 mm in diameter at 150 MPa. Finally, the binder was burnt out at 800 °C for 2 h and the pellets were sintered at 1300 °C for 2 h.

### Electric properties characterization

All samples were polished to the thickness of 0.10–0.15 mm and the silver thin film was sputtered with the diameter of 0.75 mm as electrode. The room temperature polarization-electric field (P-E) hysteresis loops were characterized by aix ACCT TF 2000 analyzer FE measurement system (aix ACCT Co., Aachen, Germany) at 10 Hz and different electric fields.

### X-ray diffraction

The X-ray diffraction were examined by an X-ray diffractometer (D/Max-2550V, Rigaku, Tokyo, Japan) with a Cu-Kα radiation. The slow scanning speed (time/step: 3 s per 0.005°) was set in order to clearly observe the superlattice reflections.

### Transmission electron microscopy (TEM)

TEM specimens were prepared by a conventional approach combining mechanical thinning and finally Ar + ion-milling in a Gatan PIPS II. The ion-milling voltage was gradually decrease from 3 keV to 0.5 keV to reduce ion-beam damage. Specimens were then coated with a thin-layer of carbon to minimize charging under the electron beams. The dark-field (DF) images and select-area electron diffraction (SAED) patterns were carried out on JEOL JEM-2100F microscope. The atomic-scale high-angle annular dark-field (HAADF) and annular bright-field (ABF) images were carried out on Cs-corrected JEOL JEM-ARM300F and Hitachi HF5000 microscopes. The experiment conditions were: probe size in 7C mode and convergence semi-angle of 18 mrad and collection semi-angle of 53–180 mrad (HAADF) and 10–20 mrad (ABF) for JEOL microscope; probe size in UHR mode and convergence semi-angle of 20 mrad and collection semi-angle of 60–320 mrad (HAADF) and 11–22 mrad (ABF) for Hitachi microscope.

The accurate and precise extraction of atomic column positions from HRSTEM images permits to quantify crystal structure parameters along the selected direction. In this case, the dipoles ordering configuration can be immediately accessible by direct imaging of the crystal structure. In this work, the picometer-precision fitting of atomic columns was done by MATLAB code, a least squares estimation algorithm for accurate and precise quantification of the atomic column positions and intensities with considering overlap between neighboring atomic columns, which has been used in previous literatures^34,35^. Considering the fact that the B-cations have very small displacement, the B-cations positions are averaged as reference points to calculate the displacement of A-cations, B-cations, and O-anions. The fitting of the atomic columns for A-cations, B-cations, and O-anions in this work has a 95% confidence interval of 4 pm, 3 pm, and 7 pm, respectively. The larger deviation of A-site columns than the one of B-site columns represents an overestimation of the precision of the fitting algorithm due to the modulated structure, which leads to a large fluctuation in distance between adjacent columns. Nevertheless, the polarization maps and profiles reveal the same evolution of modulation period with XRD (Fig. 1a), SAED (Fig. 1b and Supplementary Fig. 1) and modulation fringes (Supplementary Fig. 3), implying that the calculation results in this study are reliable and secure.

## Supplementary information

Supplementary Information

## Data Availability

The data that support the findings of this study are available from the corresponding author upon reasonable request.
