# Supplementary material for: Unveiling the ferrielectric nature of PbZrO3-based antiferroelectric materials
Source: Nat Commun. 2020 Jul 30;11:3809. doi: 10.1038/s41467-020-17664-w (PMC7392892; doi:10.1038/s41467-020-17664-w)
Supplement: Supplementary file 1 — Supplementary Information [file 41467_2020_17664_MOESM1_ESM.pdf]

Supplementary Information

Unveiling the ferrielectric nature of $PbZrO_3$-based antiferroelectric materials

Fu et al

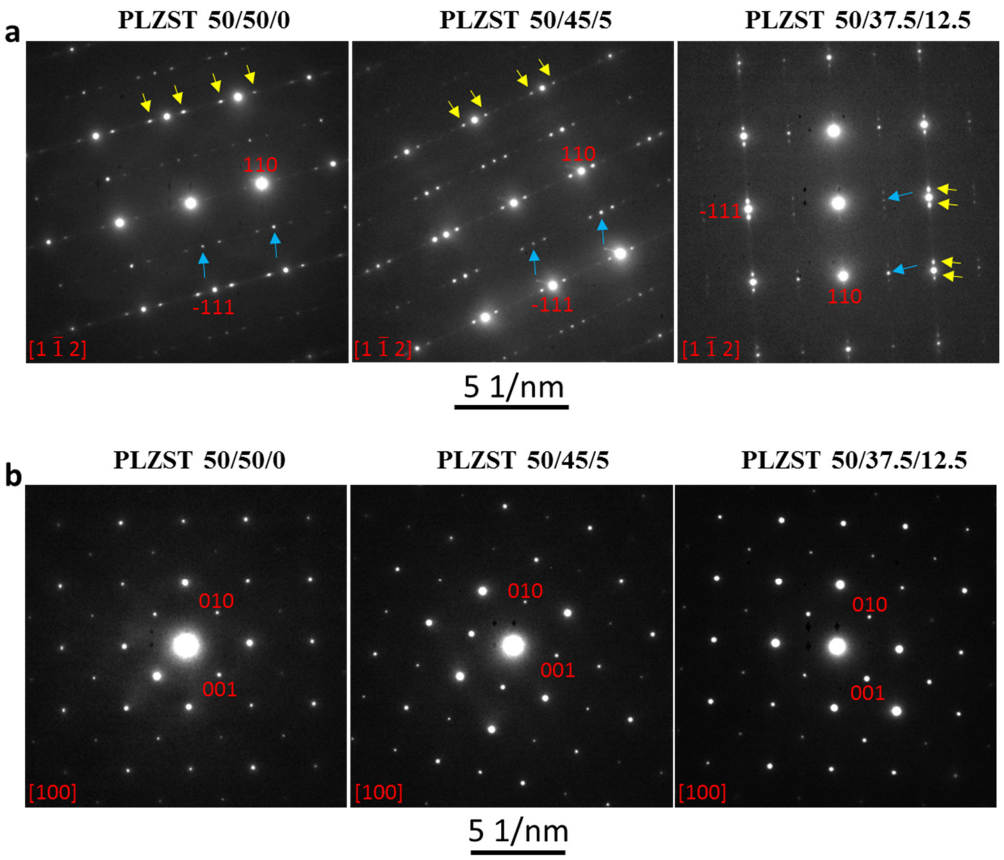

Supplementary Figure 1

(a, b) The SAED patterns of PLZST system viewed along the $[1\bar{1}2]$ and [100] direction, respectively. The satellite reflections associated with modulation mode and the superlattice reflections associated with octahedra tilt are marked by yellow and blue arrows in (a), respectively. The SAED patterns for [100] and [010] zone axes are the same and were indexed based on the [100] direction here in (b). There are no satellite reflections in [100]/[010] SAED patterns implying that the polarization component along the [001] direction is zero or a constant without modulation, which doesn't affect the ferrielectric ordering. The satellite reflections appear (see yellow arrows as examples) around each basic reflections in the SAED patterns of [001] and $[1\bar{1}2]$ zone axes (Supplementary Figure 1a and Fig. 1b) while disappear in SAED pattern of [100] zone axis (Supplementary Figure 1b), indicating that the modulated structures of PLZST system are one-dimensional along [110] direction. These satellites are located at irrational positions with respect to the interval of the $[110]^*$ sequence indicating the modulated structures are incommensurate. By examining the spacing between basic spot and the nearest satellite, it can be seen that the modulation period increase with decreasing Sn/Ti ratio (Supplementary Figure 1a and Fig. 1b). Beside the satellite reflections, the 1/2{ooo} (all h, k, l are odd) superlattice reflections (see blue arrows as examples) can also be observed in the SAED patterns along $[1\bar{1}2]$ direction (Supplementary Figure 1a), which refer to the existence of antiphase tilt of oxygen octahedra. The intensity of 1/2{ooo} gradually decreases with decreasing Sn/Ti ratio implying structural evolution of oxygen octahedra with composition.

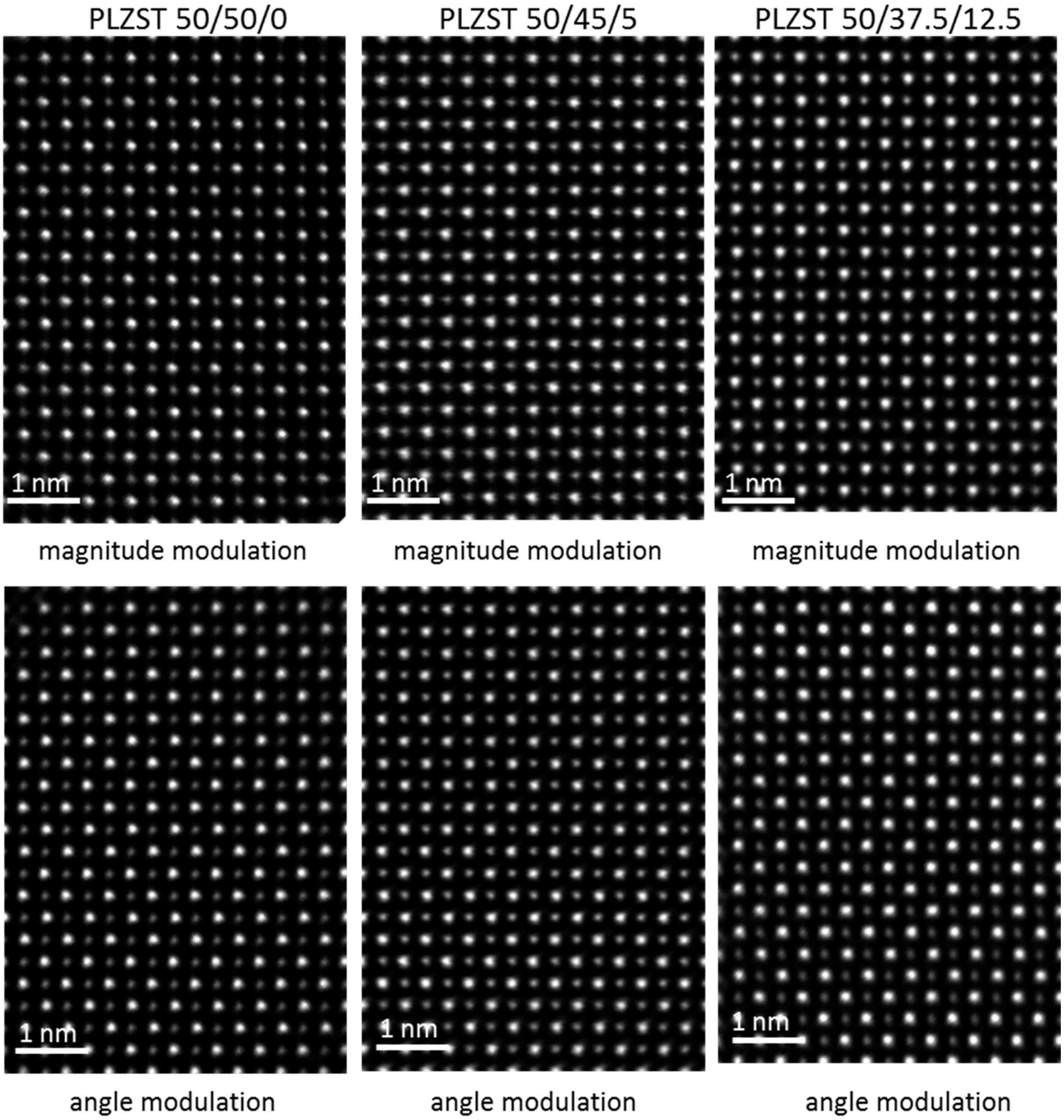

Supplementary Figure 2

The full view of atomic-scale HAADF images of PLZST system in Fig. 2. The large and small dots refer to A-site and B-site atomic columns, respectively.

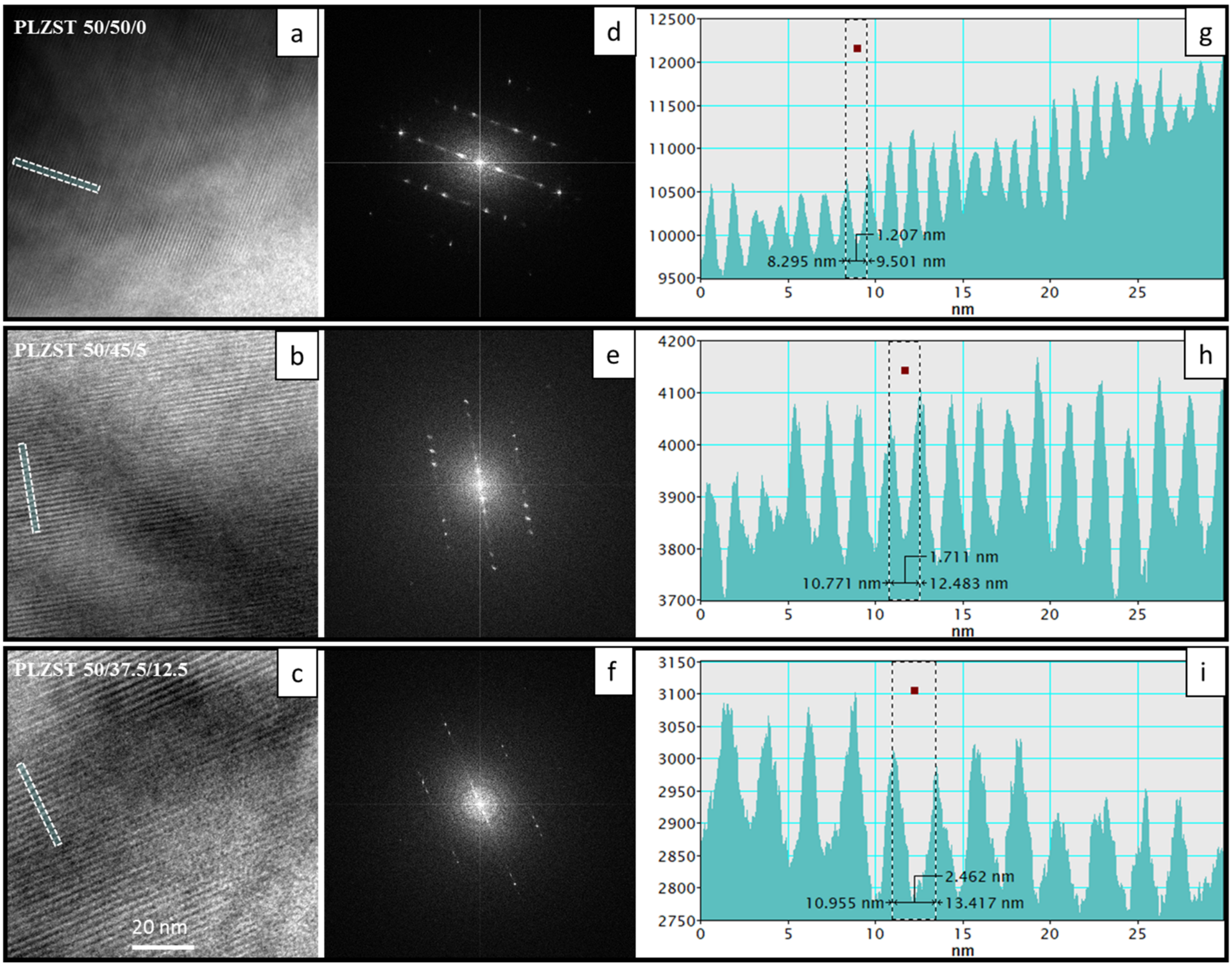

Supplementary Figure 3

The medium-magnified TEM images (a, b, c) and corresponding fast Fourier transform (d, e, f) of PLZST samples viewed along the [001] direction. The coherent fringes are resulted from the modulation of dipoles. The line-scan profiles (g, h, i) of corresponding TEM images clearly show the modulation period, which is consistent to the mapping of atomic displacements.

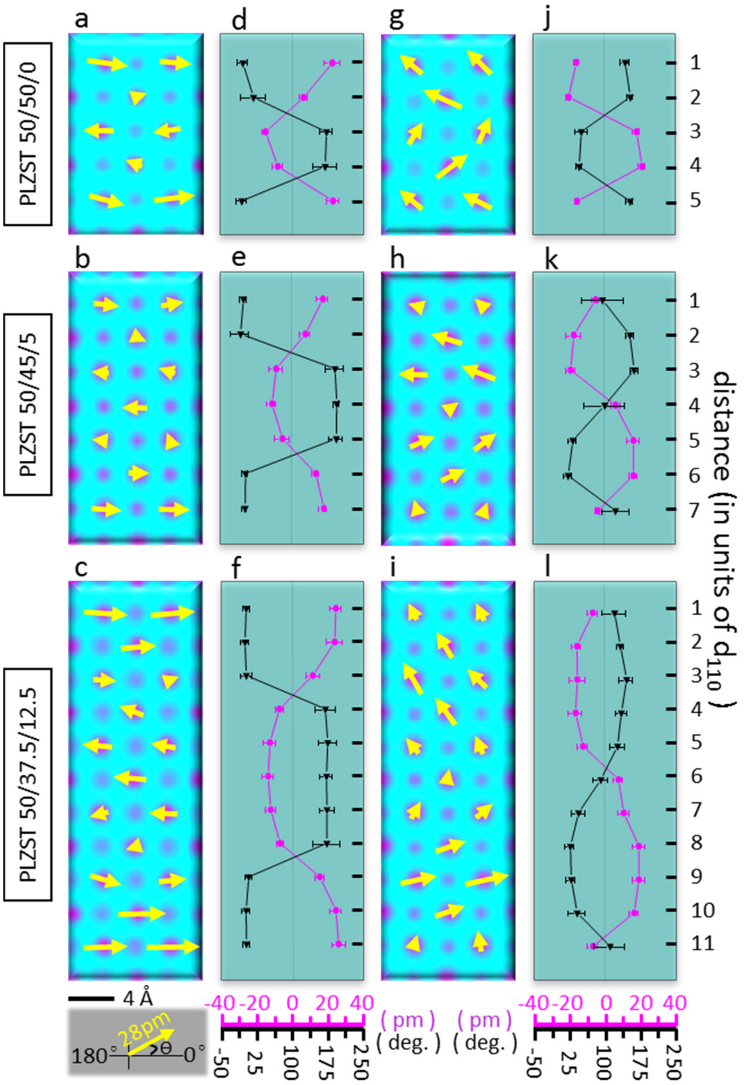

Supplementary Figure 4

Experimental unit cells of (a, b, c) magnitude modulation mode and (g, h, i,) angle modulation mode of PLZST system. Because of the fluctuated period and the disorder of incommensurate structure, the unit cells are just picked for showing the structural characteristic. For clarity, the atomic-scale HAADF images are false-colored and superimposed with displacement vectors. (d, e, f, j, k, l) Magnitude (purple dots and lines) and angle (black dots and lines) of the average atomic displacement vectors at each dipoles line in the corresponding unit cells. These quantitative data are profiled by compiling statistics on tens of isomorphic unit cells as presented in Supplementary Figure 4, where the magnitude of $\mathbf{D}_{\mathbf{AB}}$ is defined as negative value when it aligns to the left. Cleary, the modulation waves can be immediately accessible to allow the direct visual comparison of the modulation amplitude and period. Of particular significance is that the value of positive magnitude is larger than the absolute value of the negative one in magnitude modulation mode while they are nearly equal in the angle modulation mode. In such case, the net polarization will produce in both magnitude (in horizontal direction) and angle (in vertical direction) modulation modes. The error bars represent the standard deviation measured from the experimental image.

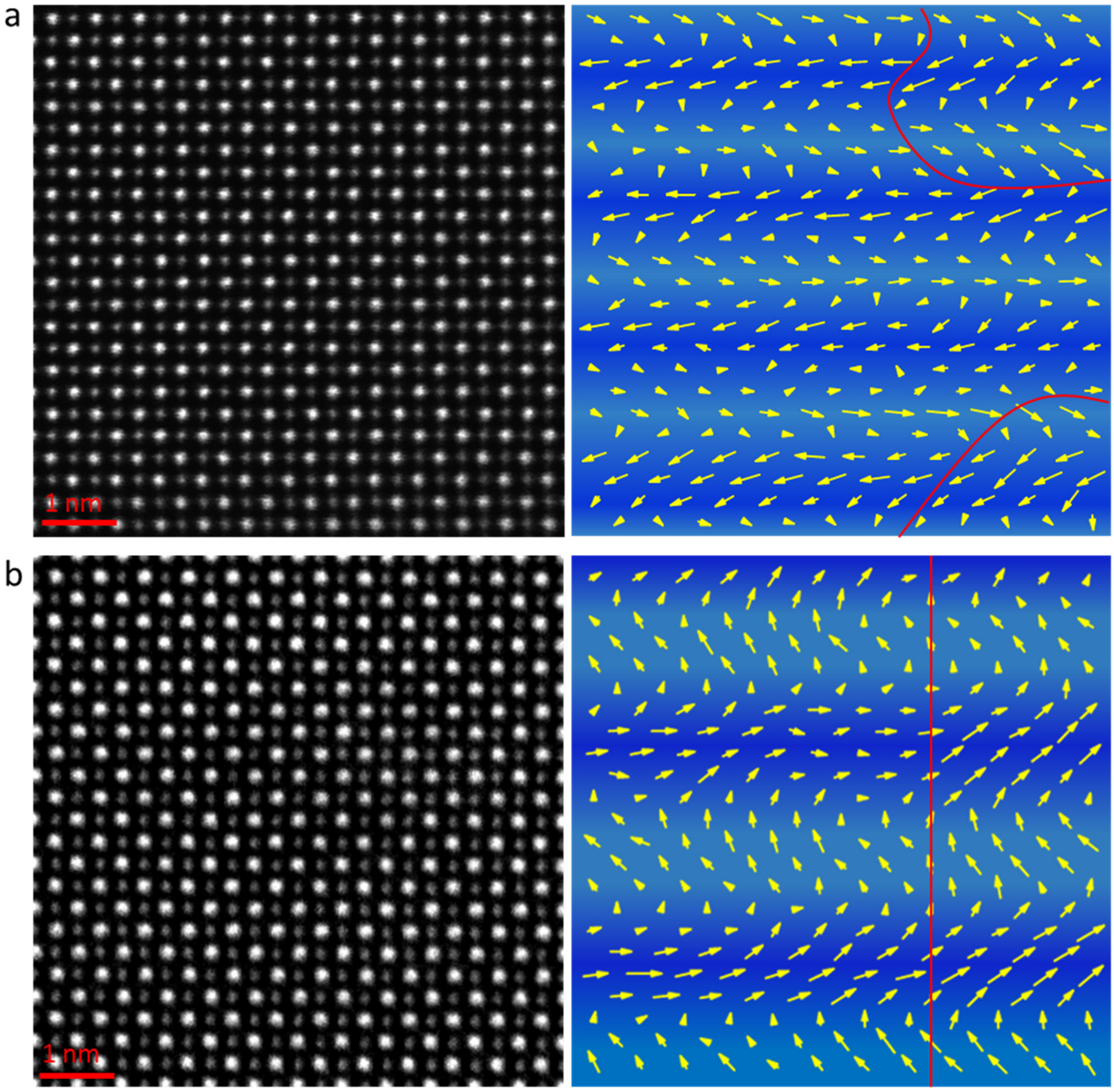

Supplementary Figure 5

The atomic-scale high-angle annular dark-field (HAADF) image and corresponding displacement vectors of A-site cations in PLZST 50/45/5 (a) and PLZST 50/37.5/12.5 (b). The two displacement mappings are examples to show the coexistence of magnitude modulation mode and angle modulation mode, whose boundary are outlined approximately by red line.

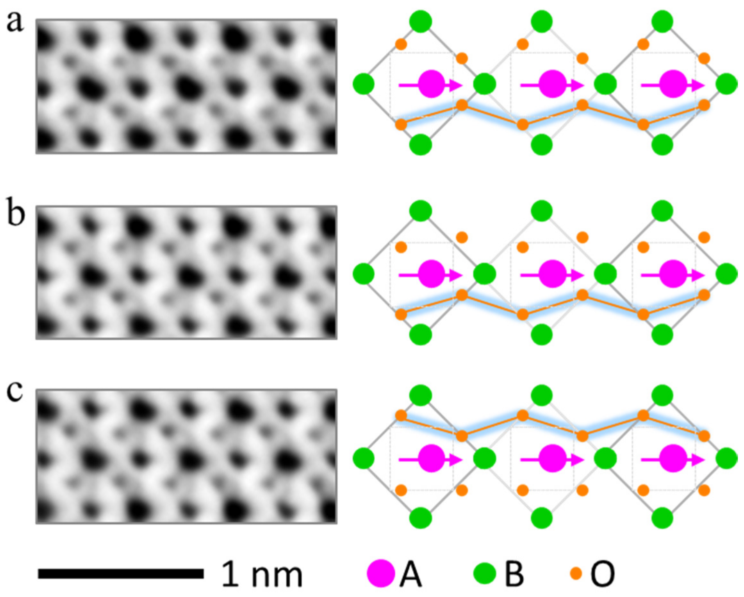

Supplementary Figure 6

The magnified ABF images and corresponding ideal model of atomic displacement in PLZST 50/50/0. (a, b, c) show three different y-rippling behaviors of two adjacent O-rows around the A-row with large displacement, respectively. It can be seen that the larger displacement of A-cations will result in stronger repulsive force between A-cations and its pointed B-cations. In this case, there is at least one of its neighbouring O-rows (highlighted by zigzag line) configuring high degree of y-rippling to weaken the repulsive force.

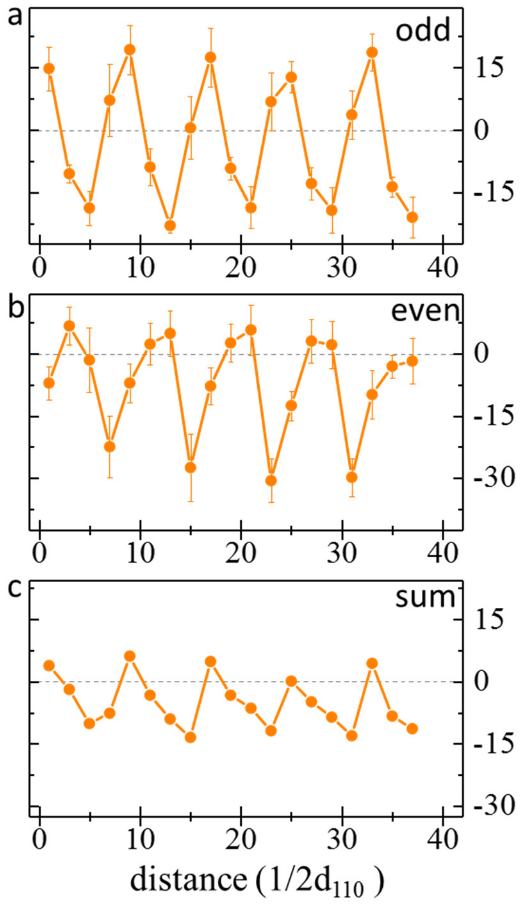

Supplementary Figure 7

The horizontal displacement of odd O-column (a), even O-column (b) and sum of two types O-columns (c) in Fig. 3b. The profile of odd O-column is presented in Fig. 4b, rather than the sum profile, to reveal the displacement amplitude of O-anions. The error bars represent the standard deviation measured from the experimental image.

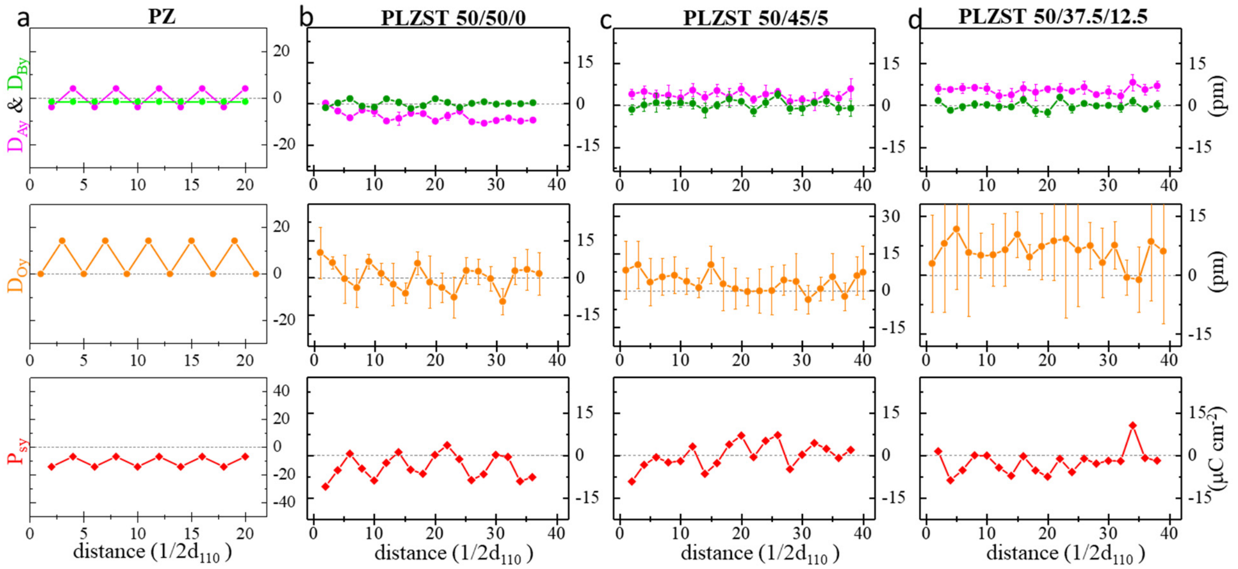

Supplementary Figure 8

(a-d) The vertical component of atomic displacement and spontaneous polarization for each atomic row is plotted as a function of distance along the y-direction for $PbZrO_3$ and PLZST system. The purple, green, orange and red profiles represent the A-cations, B-cations, O-anions and $\mathbf{P_s}$, respectively. The values of these profiles are calculated from the Figs. 3a~3d, where the displacement are defined as negative value when it aligns to the up. The O1 and O2 located at (001) face center of ideal perovskite unite cell are not considered in $PbZrO_3$ because they are overlaid with B-cation column in experimental ABF images of PLZST system. The vertical $\mathbf{P_s}$ will not be discussed in the main text of this paper because it has relatively small value and large errors. Meanwhile, the vertical $\mathbf{P_s}$ has no influence on the dipole modulation and ferrielectric configuration. Note the all figures have the same scales with Fig. 4. The error bars represent the standard deviation measured from the experimental image.

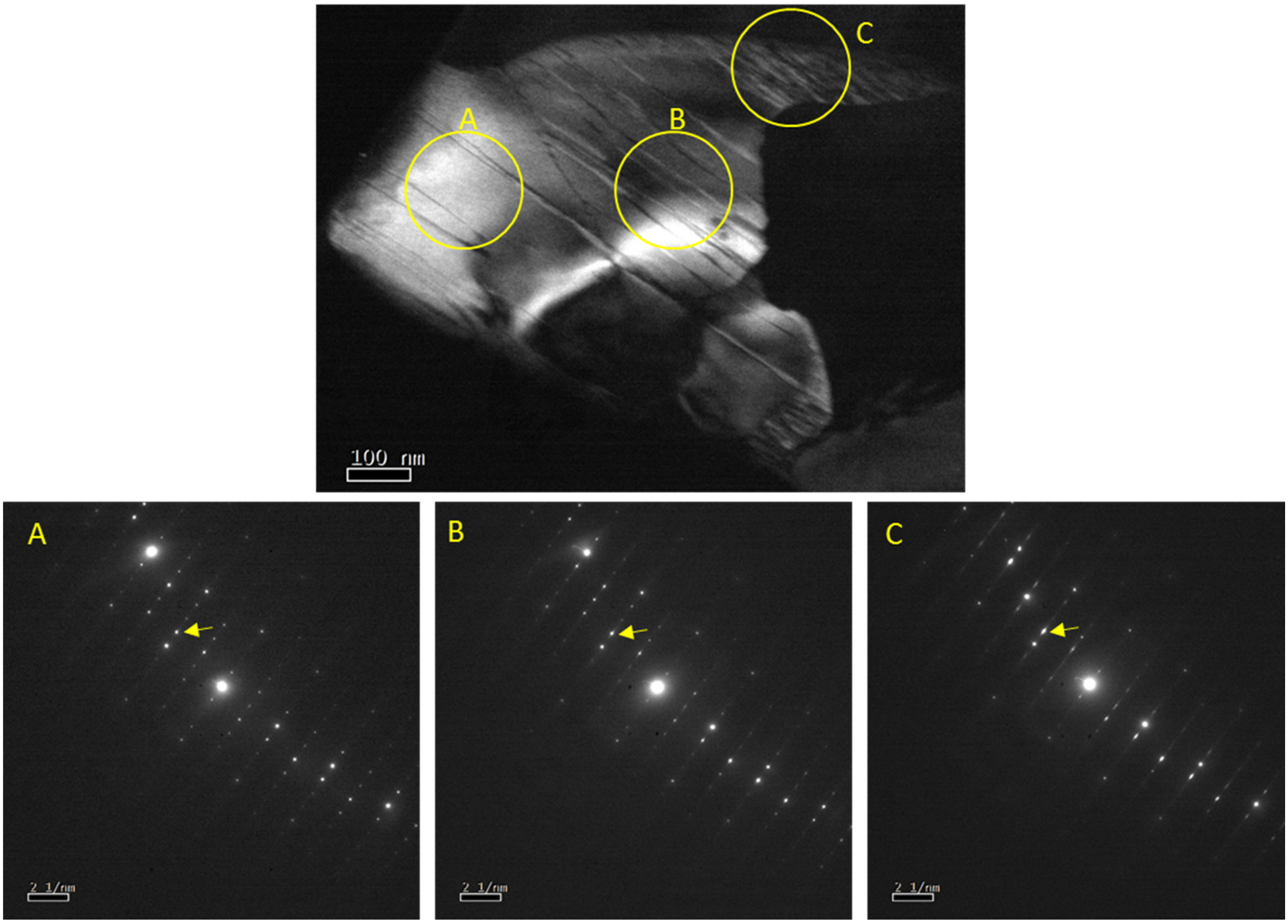

Supplementary Figure 9

The coexistence of AFE and FiE ordering in PLZST 50/50/0 ceramics. According to our experience, the SAED and dark-field imaging are alternative methods which can achieve more average statistic to study the dipoles ordering. Here, the SAED shows the superlattice reflections with a quarter of d_{110} (marked by yellow arrow) change from circular spots to elongated one, and the corresponding dark-field image presents that the antiphase boundaries change from low-density to high-density. The evolution of SAED and dark-field image suggests the gradually change from AFE dipoles ordering (ordered 4-fold modulation period) to FiE dipoles ordering (disorder and deviation from 4-fold modulation period) from area A to area C in a single grain.

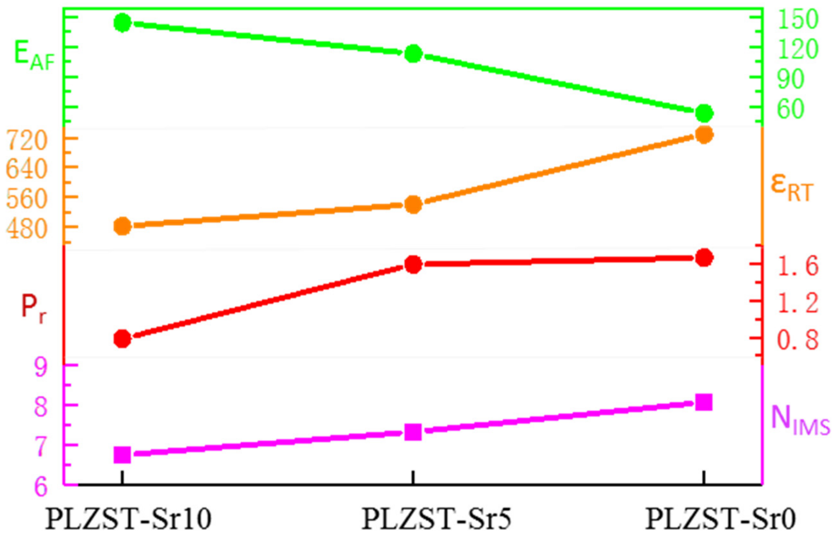

Supplementary Figure 10

Modulation period (N_{IMS}), remanent polarization (P_r, in unit of μC cm^{-2}), dielectric constant (ε_{RT}) and forward switching field (E_{AF}, in unit of kV cm^{-1}) of $(Pb_{0.97-x}Sr_xLa_{0.02})(Zr_{0.60}Sn_{0.30}Ti_{0.10})O_3$ system. The change trend of electric properties as a function of N_{IMS} is consistent with the PLZST system.

Supplementary Table 1

The detailed numerical results of N_{IMS}, P_r, ε_{RT} and E_{AF} in PLZST system.

sample \ parameters	N_{IMS}	P_r (μC cm^{-2})	ε_{RT}	E_{AF} (kV cm^{-1})
PLZST 50/50/0	4.04	1.23	222	246
PLZST 50/45/5	6.15	2.71	393	133
PLZST 50/37.5/12.5	9.37	3.77	911	13

Supplementary Table 2

The detailed numerical results of N_{IMS}, P_r, ε_{RT} and E_{AF} in Sr-doped PLZST system.

parameters / sample	N_{IMS}	P_r ($\mu C\ cm^{-2}$)	ε_{RT}	E_{AF} ($kV\ cm^{-1}$)
PLZST 50/50/0	6.74	0.79	482	144
PLZST 50/45/5	7.32	1.60	541	113
PLZST 50/37.5/12.5	8.07	1.67	729	53
